# LSEC model of aging

**DOI:** 10.18632/aging.103492

**Published:** 2020-06-13

**Authors:** Laurent Grosse, Dmitry V. Bulavin

**Affiliations:** 1 Institute for Research on Cancer and Aging of Nice (IRCAN), INSERM, Université Côte d’Azur, CNRS, Nice, France

**Keywords:** aging, liver sinusoid endothelial cells, senescencee, lifespan

## Abstract

Data obtained from genetically modified mouse models suggest a detrimental role for p16^High^ senescent cells in physiological aging and age-related pathologies. Our recent analysis of aging mice revealed a continuous and noticeable accumulation of liver sinusoid endothelial cells (LSECs) expressing numerous senescence markers, including p16. At early stage, senescent LSECs show an enhanced ability to clear macromolecular waste and toxins including oxidized LDL (oxLDL). Later in life, however, the efficiency of this important detoxifying function rapidly declines potentially due to increased endothelial thickness and senescence-induced silencing of scavenger receptors and endocytosis genes. This inability to detoxify toxins and macromolecular waste, which can be further exacerbated by increased intestinal leakiness with age, might be an important contributing factor to animal death. Here, we propose how LSEC senescence could serve as an endogenous clock that ultimately controls longevity and outline some of the possible approaches to extend the lifespan.

##  

The accumulation of senescent cells has been shown detrimental in many contexts [1–5]. In turn, the analysis of senescence in vascular endothelial cells is one of the main directions in the field of vascular aging as it plays a key role in the initiation, progression, and advancement of cardio-vascular diseases [3, 6–9]. While attenuating senescence has already been shown to ameliorate several pathological conditions, more recent work suggested that elimination of senescent cells could be advantageous for health and lifespan [4, 10–12]. Removing certain senescent cells that can be robustly replaced without conferring changes on organ structure or function is clearly beneficial. However, accumulating data suggest that there are functionally important senescent cell types that might not be efficiently replaced under physiological conditions, especially in old organisms. For example, age-induced senescence has been recently described in hypothalamic stem cells [13], while we found a significant build-up of senescence in liver sinusoid endothelial cells (LSECs).

LSECs are fenestrated endothelial cells that line the hepatic sinusoids. These cells have several important physiological roles, including facilitating the bidirectional transfer of substrates between the blood and hepatocytes, endocytosing circulating proteins, regulating immunotolerance, and maintaining sinusoidal microenvironment [14–24]. LSECs are the main cell type responsible for clearing blood-borne macromolecular waste [14–16], including most viruses [17–19] and lipopolysaccharides (LPS) [20, 21]. Furthermore, LSECs are responsible for the selective uptake of high-density lipoprotein [20, 22], in this way governing cardiovascular risk and all-cause mortality [23, 24]. Among the many toxins that are removed by LSECs, oxidized low density lipoprotein (oxLDL) [25, 26] is of specific interest as a major atherogenic substance [27–29]. Other toxic agents endocytosed by LSECs include Advanced Glycation End products (AGEs) — heterogenous metabolic by-products formed by non-enzymatic irreversible protein glycosylation/glycoxidation and that are resistant to proteolysis [30–32]. The accumulation of AGEs in tissues is harmful, observed in several pathological conditions [33–35], and thought to result from chronic hyperglycemia and increased oxidative and carbonyl stress [36–38]. The removal of both oxLDL and AGEs, however, is an inefficient process and their build up, as seen in several pathological conditions or after a depletion of LSECs [39], could rapidly overwhelm the LSEC clearing capacity. This in turn could cause further oxidative stress and has significant negative impact both in liver sinusoids and extrahepatic vascular beds [25, 40–42].

There are substantial age-induced changes in the structure and function of LSECs which in turn impact liver functions contributing to hepatic insulin resistance but also have a systemic risk of cardio-metabolic diseases [29, 30, 43–45]. Age-induced morphological changes in LSECs have been described in several species including humans [46], rats [47], baboons [48], and wild-type [30] and genetic mouse models of premature aging [49]. These morphological changes are characterized by pseudocapillarization, which includes defenestration (a reduction in the number and size of fenestrations), endothelial thickening, and basal lamina and collagen deposition [14, 50, 51]. Age-induced pseudocapillarization is a sequential process, starting with defenestration at a relatively young age and progressing towards LSEC thickening and fibrosis later in life (Figure 1) [30]. In turn, finding approaches to reverse defenestration and pseudocapillarization have being actively and successfully pursued in several laboratories as a strategy to ameliorate some age-related diseases [52–55].

**Figure 1:**
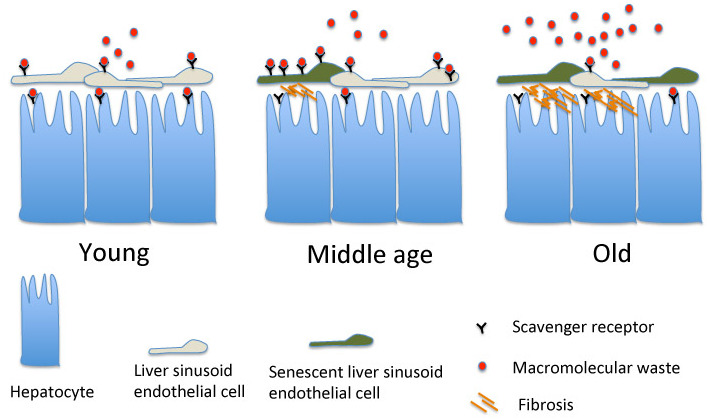
**LSEC senescence throughout animal lifespan.** The accumulation of p16^High^ senescent LSECs starts gradually and is characterized by an increased expression of scavenger receptors (SRs) and endocytic activity in middle (1-year) age animals. This increase well-compensates for the loss of clearing functions by hepatocytes potentially due to LSEC defenestration. Later in life, however, the expression of SRs and LSEC endocytic activity are significantly reduced resulting in build up of blood-born macromolecular waste. This in turn contributes negatively to lifespan.

Pseudocapillarization is accompanied by changes in the expression of multiple genes, of which some are associated with senescence. Accumulated p16 senescence marker expression, elevated mitochondrial oxidative stress and increased expression of inflammatory genes resembling the senescent secretome have been recently reported in LSECs [30, 47]. Furthermore, LSECs in old animals are considered to be in a moderate pro- inflammatory state [30, 47, 51]. We further extended this analysis and found that numerous markers of senescence are continuously increased in mouse LSECs with age (Figure 1) [39]. These findings unambiguously confirm that LSECs undergo aging-induced senescence, which in turn could be a part of the pseudocapillarization process. The way in which senescence and pseudocapillarization are interconnected however warrants further analysis.

Both LSECs [14, 21] and hepatocytes [56] express numerous receptors that are essential for removing different macromolecules from the bloodstream. Any deregulation to this process could trigger compensatory mechanisms [57]. If access between the blood content and hepatocytes is blocked, as occurs during age-induced LSEC defenestration [30, 47, 58], this could trigger a compensatory upregulation in SR expression on the LSEC surface. This effect would in turn result in enhanced oxLDL and AGE intake, further inducing oxidative stress and eventually senescence.

In our own analyses of middle-aged mice (1 year old), we observed a substantial increase in SR expression on p16^High^ senescent LSECs, further supporting this mechanism as a starting point for LSEC senescence [39]. Increased SR expression on early senescent cells fuels a further intake of toxic substances, thus creating a positive feedback loop to drive mitochondrial oxidative stress and deeper senescence. Once senescence progresses, it triggers heterochromatin silencing, which in turn can suppress the expression of numerous SRs and endocytosis genes to reduce endocytic LSEC activity both *in vivo* and *in vitro* [31, 32]. A downregulation in LSEC endocytic activity was previously observed in old rats and mice when using FSA/BSA and AGEs as substrates [30–32]. A “traffic jam hypothesis” was put forward to explain this downregulation: here, the decreased LSEC endocytic capacity in old animals is due, at least in part, to increased endothelial thickness, which in turn slows down the transport of internalized ligands to endo/lysosomal compartments [32]. Our recent results argue that reduced LSEC endocytic activity with older age could also be a consequence of transcriptional suppression that is common to senescent cells due to widespread heterochromatin silencing (Figures 1, 2) [59]. In fact, some SRs are indeed downregulated in older animals [39, 47, 51]. It would be important, however, to investigate the level of expression of SRs and endocytosis genes in very old animals (for example in 2.5-3 year-old mice) where the mechanism of senescence-induced transcriptional silencing could be especially relevant. Ultimately, the inability of LSECs to clear numerous dangerous substances from the blood could be a significant contributing factor to various age-related pathologies [58]. Furthermore, an accumulation of macromolecular waste and toxins, which is further fueled by increased intestinal permeability with age [60, 61], could create a “death cross” (Figure 2) leading to the loss of the animal once the level of clearance drops below the threshold required for survival.

**Figure 2:**
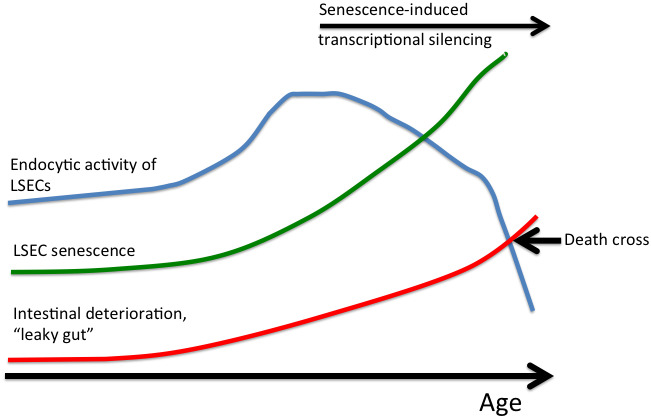
**LSEC senescence as an endogenous aging clock.** Senescent LSECs loose the ability to clear numerous dangerous substances from blood resulting in an age-induced accumulation of macromolecular waste and toxins. This in turn is further exacerbated by increased intestinal permeability, which induces further increase in the level of endogenous toxins. Once the level of clearance drops below the threshold required for survival (“death cross”), the animal dies.

Based on proposed model, several approaches could be considered to target senescent LSECs in order to ameliorate some age-related diseases and potentially to extend lifespan.

### Delaying LSEC senescence

A list of drugs that might efficiently block and/or delay senescence is still in the making. However, possible candidates could come from the ongoing analysis of compounds that reduce or reverse LSEC defenestration, as both events seem to be interconnected *in vivo*. A recent study indicated that by targeting the nitric oxide pathway and inducing actin remodeling, it is possible to attenuate defenestration in old mice [52]. In another study, activating AMPK signaling and autophagy with either acute fasting (which increases the diameter of fenestrations) or chronic caloric restriction over a lifetime reversed the age-related loss of fenestrations [62]. In a similar manner, metformin, which acts on the AMPK nutrient-sensing pathway [55, 63], increased fenestration porosity in old mice and improved insulin sensitivity [53]. Finally, there have been reports that resveratrol, which acts on the sirtuin-mediated nutrient-sensing pathway [64], increases fenestrations in a Werner Syndrome mouse, which is a model for premature aging [65]. While many of the above-mentioned compounds could potentially attenuate LSEC senescence *in vivo*, to achieve their maximal effect, these compounds should be taken continuously which somewhat diminishes their overall utility.

### Reversing transcriptional silencing of SRs

It is not fully understood how enhancer chromatin, epigenetic marks, transcription factor recruitment, and the organizational principles of transcription factor networks drive the senescence program. Recent studies showed that the senescence program is predominantly encoded at the enhancer level [66, 67] and that the enhancer landscape is dynamically reshaped at each step of the senescence transition [68]. A deeper understanding of the underlying mechanisms might help identify ways in which enhancer regulation could be manipulated to overcome potential SR and endocytosis gene transcriptional silencing in senescent LSECs, which express enhanced levels of heterochromatic marks such as macroH2A and Histone H9Me2 [39]. In turn, reversing the transcriptional suppression of several SRs and endocytosis genes in the later stages of LSEC senescence could contribute positively to lifespan.

### Reprogramming senescent LSECs

Another approach to target and rejuvenate LSECs could lie in senescence reprogramming [67, 69]. This approach represents a broader direction that is not restricted just to reversing transcriptional silencing of SRs but to changes of entire transcriptional network to a younger non-senescent state. Pioneer transcription factors (that directly bind condensed chromatin) are critical in establishing new cell fate competence: they grant long-term chromatin access to non-pioneer factors and help determine cell identity by opening and licensing the enhancer landscape [68, 70, 71]. In addition, DNA methylation could play an important role in establishment of senescence as recently was shown for a DNMT1-dependent downregulation of BRCA1/ZNF350/RBBP8 repressor complex in the course of oncogene-induced senescence [72]. Thus, efforts to identify different factors and the signaling pathways that they control could be critical in understanding the feasibility of rejuvenating senescent LSECs as a therapeutic strategy.

### Removing and replacing senescent LSECs

Removing and replacing senescent LSECs is perhaps the most challenging approach of all those described here. LSEC removal will instigate an immediate fibrotic response [39], which must be suppressed before any active replacement mechanism takes place. Furthermore, replacement of senescent LSECs seem to be challenging and could be divided into (i) stimulating the proliferation of remaining LSECs (perhaps via the hyperactivation of VEGF signaling) [73] or (ii) repopulating damaged sites with hepatic and extrahepatic LSEC progenitors [74]. The latter approach however requires further investigation, as the nature of LSEC progenitors is highly debated.

### Preventing an age-induced “leaky gut”

Although not directly related to targeting senescent LSECs, finding ways to ameliorate intestinal health to improve health and extend the lifespan seem justified [61, 75]. An increasingly leaky intestine that ultimately increases the toxic load with age will counteract any benefits of blocking LSEC senescence. While the approaches to pharmacologically address this problem are still incomplete, some dietary recommendations could be considered. For example, there is emerging evidence that heavy alcohol use, stress and even the Western pattern diet, which is low in fiber and high in sugar and saturated fats, might initiate intestinal deterioration [75, 77]. As such, balancing your diet and controlling your gut flora could not only improve your intestinal health but also positively contribute to the efforts of reducing the senescent LSEC load.

Identifying the full repertoire of senescent cells *in vivo* is critical in understanding how their removal might affect a healthy lifespan. There is no doubt that eliminating some senescent cells is beneficial for healthy aging and overall lifespan [78–80]. However, there are abundant p16^High^ senescent cell types in the aging organism that are structurally and functionally important while their removal could have detrimental consequences. Specifically, we recently showed that senescent LSECs are not replaced by non-senescent neighbors, but instead their removal activates another type of regenerative response — fibrosis [39]. As such, non-selective senescent cell removal should be considered with great caution as it could have a serious negative health impact in older organisms. This problem however could be partially solved by using drugs that selectively remove defined senescent cell types. While such selective elimination could be beneficial in age-related diseases, this approach as a life-extension strategy has its limitations as non-removed senescent cells will continue to accumulate with age, ultimately debilitating the organism. We recently found that LSECs undergo a noticeable aging-induced senescence in mice and this provides significant advantages in terms of their targeting due to a relatively easy accessibility and high endocytic activity. We thus propose that delaying senescence, reprogramming or replacing senescent LSECs could represent a powerful tool to retard aging.
